# Identification of Salt Stress Biomarkers in Romanian Carpathian Populations of *Picea abies* (L.) Karst.

**DOI:** 10.1371/journal.pone.0135419

**Published:** 2015-08-19

**Authors:** Sorin T. Schiop, Mohamad Al Hassan, Adriana F. Sestras, Monica Boscaiu, Radu E. Sestras, Oscar Vicente

**Affiliations:** 1 Department of Forestry, Faculty of Horticulture, University of Agricultural Sciences and Veterinary Medicine, Cluj-Napoca, Cluj, Romania; 2 Institute for Plant Molecular and Cellular Biology (IBMCP, UPV-CSIC), Universitat Politècnica de València (UPV), València, Spain; 3 Mediterranean Agroforestal Institute (IAM), Universitat Politècnica de València (UPV), València, Spain; 4 Department of Horticulture and Landscaping, Faculty of Horticulture, University of Agricultural Sciences and Veterinary Medicine, Cluj-Napoca, Cluj, Romania; Estación Experimental del Zaidín (CSIC), SPAIN

## Abstract

The Norway spruce (*Picea abies*), the most important tree species in European forests, is relatively sensitive to salt and does not grow in natural saline environments. Yet many trees are actually exposed to salt stress due to the common practice of de-icing of mountain roads in winter, using large amounts of NaCl. To help develop strategies for an appropriate use of reproductive seed material on reforestation sites, ensuring better chances of seedling survival in salt-affected areas, we have studied the responses of young spruce seedlings to salt treatments. The specific aim of the work was to identify the optimal salt stress biomarkers in *Picea abies*, using as experimental material seedlings obtained by germination of seeds with origin in seven populations from the Romanian Carpathian Mountains. These responses included general, conserved reactions such as the accumulation of ions and different osmolytes in the seedlings needles, reduction in photosynthetic pigments levels, or activation of antioxidant systems. Although changes in the contents of different compounds involved in these reactions can be associated to the degree of stress affecting the plants, we propose that the (decreasing) levels of total phenolics or total carotenoids and the (increasing) levels of Na^+^ or K^+^ ions in *Picea abies* needles, should be considered as the most reliable and useful biomarkers for salt stress in this species. They all show very high correlation with the intensity of salt stress, independently of the genetic background of the seeds parental population, and relatively easy, quantitative assays are available to determine their concentrations, requiring simple equipment and little amount of plant material.

## Introduction

Worldwide, the land occupied by either saline or sodic soils is estimated as close to 10^9^ ha [1]. In the next decades, according to forecasted changes in temperature and precipitation patterns, soil salinisation will intensify [2]. Warm and dry climate will lead to an increase in salt accumulation, seepage and wind deposition, while the salts already scattered in the soil will become more concentrated [3,4]. Other sources of increasing salt concentration in the soil could derive from irrigation water and rock decomposition, inorganic fertilizers or escalation of global deforestation [5,6].

Plant responses to salt stress involve different physiological traits, molecular networks and biochemical pathways that affect a wide range of basic processes at the cell and whole-plant levels, such as ion transport and homeostasis, water relations, osmotic adjustment, or growth regulation [7–9]. High soil salinity decreases respiration rates and causes unbalance of cellular ions, changing the K^+^: Na^+^ ratio and leading to production of free radicals and other toxic reactive oxygen species (ROS) [10–12]. The effects of salt exposure in plants include a quick osmotic phase, causing low water absorption; while at longer times ion toxicity and interference with mineral nutrition alter carbon balance [13, 14].

Natural plant populations are supposed to have slowly adapted to their specific habitats, so that quick changes in environmental conditions—such as an increase in the level of abiotic stress derived from the foreseeable effects of climate change or from more direct human interventions—will most likely have a negative effect on their growth and reproductive success; yet the differences in the genetic background of distinct, isolated populations of the same species should provide some flexibility in the response to the more intense or new stressful conditions [15]. Therefore, the success of descendants' acclimatisation to altered habitats might be correlated with the climatic conditions of the region of origin; this can be an useful tool in screening for plants that may respond better to short or long-term exposure to stress. Biochemical indicators of salt tolerance in plants are very diverse, but the identification of species-specific markers is of great relevance for plant breeding [16]. On the other hand, studies on the responses of different species to salt stress may provide new directions in applied technology, especially in plant reproduction processes.

*Picea abies* (L.) Karst. (the Norway spruce) is the most important tree species of Europe's forests. It widely ranges from the northern to the southern parts of Europe’s forested lands, covering more than 30 million hectares, which corresponds to 38% of the continent's coniferous area [17]. In Romania, the species covers approximately 23.4% of the forested area and represents 77% of the country's conifers [18].

Conifers show a relatively high sensitivity to salt and do not grow in natural saline environments [19]. In Europe's Norway spruce habitats, roadside areas occupied by young spruce stands are common, especially as planted windbreaks. Across the continent, anthropogenic winter activities, such as ice-removing on mountain roads using NaCl, threaten tree growth. Previous reports have shown that de-icing salt caused an annual tree death rate of over 700,000 trees in Western Europe alone [20]. According to a European study on winter maintenance practices, published more than ten years ago, the annual amount of salt used world-wide for de-icing was estimated in more than 66 million tons, and in more than 100,000 tons in Romania alone [21]; it seems correct to assume that these values have increased significantly in the intervening years. Norway spruce stands placed several hundred meters away from the roads can be affected by de-icing salt, due to several transport pathways like direct spraying, groundwater circulation, atmospheric deposition or wind [22].

Since the effects of abiotic stress in plants with slow growth rate are evident only after a prolonged period of time, we have used several biochemical markers to assess the responses of young spruce seedlings to salt stress. The seedlings were obtained by germination of seeds with origin in seven Norway spruce populations from the Romanian Carpathian Mountains, so that we could check whether the responses to salt stress were similar in all plants, or on the contrary differed according to their parental populations. The aim of the work was to select the optimal salt stress indicators in *Picea abies*, associated with its general responses to salinity at the seedling stage. Once these stress markers are identified, they can be quickly determined in salt-treated seedlings, using simple, non-destructive and quantitative biochemical assays requiring low amounts of plant material—as an alternative to more time consuming growth inhibition assays. In this way, relatively large numbers of Norway spruce seedlings could be easily screened in the nursery, to evaluate their relative salt resistance and select those with better chances of survival in salt-affected areas. Therefore, the results of this work may eventually allow an appropriate use of reproductive seed material on reforestation sites, helping to develop strategies to preserve stands exposed to salinity by de-icing of mountain roads, a common practice in Central Europe.

## Materials and Methods

### Plant material and stress treatments

**Fig 1:**
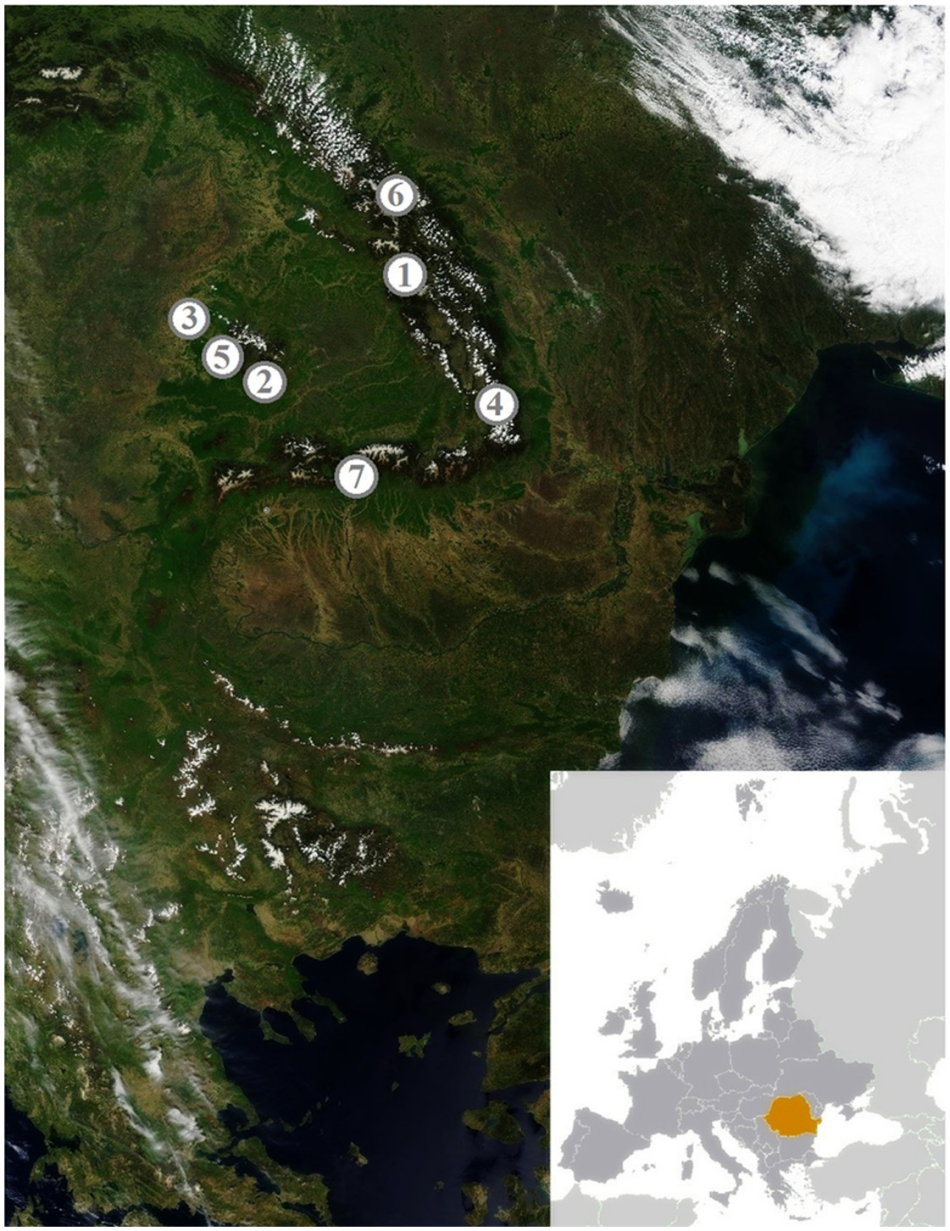
Geographical location of the origin regions for the seven studied *P*. *abies* populations. Populations are identified by numbers, used throughout the text, as follows: 1- Population Gioristea Calimanut, 2- Population Valea Mare, 3- Population Sudrigiu, 4- Population Basca Mica, 5- Population Albac, 6- Population Paraul Turculet, 7- Population Jepi. Figure modified from the U.S. CIA Romania figures (https://www.cia.gov/library/publications/the-world-factbook/index.html) and is for representative purposes only.

Seeds of Norway spruce from seven Romanian locations were obtained from Romanian gene reserve forests and seed stands included in National Catalogues of Forest Genetic Resources and Forest Reproductive Materials; the geographic locations of the populations of origin certified by the Forest Research and Management Institute Brasov, Romania are shown in Fig 1, and their respective meteorological data in Table 1. The seeds had been sown directly into moistened peat and watered with tap water for one year in a greenhouse under controlled conditions (mean temperature 21°C, relative humidity 50%) in Albac, Romania (46°27′08″ N, 22°57′05″ E), before being transferred to the greenhouse of the Institute for Plant Molecular and Cellular Biology (IBMCP), Valencia, Spain, where they were introduced into 0.5 liter pots (Ø = 11 cm) containing peat, to undergo the planned salt treatments under the following conditions: long-day photoperiod (16 hours of light), with light intensity of 130 μE m^-2^ s^-1^, temperature (23°C during the day and 17°C at night), CO_2_ level (≈ 300 ppm). Humidity ranged between 50–80% during the time course of the treatments (6 weeks).

**Table 1:** Average meteorological data of the populations under study (National Meteorological Administration, Romania, reference period 1994–2013).

Populations (Identification n°)	Altitude (m.a.s.l.)	Mean annual Temperature (°C)	Mean annual Precipitation (mm)	Mean potential ETP (mm)
**1**	**840–1200**	**6.4**	**642.0**	**558.5**
**2**	**1200–1450**	**5.6**	**893.0**	**517.3**
**3**	**230**	**10.4**	**751.9**	**672.6**
**4**	**1250–1500**	**1.9**	**800.7**	**428.6**
**5**	**770**	**7.8**	**837.6**	**594.0**
**6**	**850–930**	**7.2**	**707.8**	**607.3**
**7**	**1350–1650**	**0.9**	**1303.2**	**360.4**

Salt stress treatments were started after 14 days of acclimatization using four different concentrations of salt (0, 75, 150 and 300 mM NaCl). The control plants were watered twice a week with a standard nutritive solution in tap water (1.5 l for each tray containing 7 pots), and salt stressed plants with the same volume of the nutritive solution containing the aforementioned NaCl concentrations.

### Soil analysis

Electrical conductivity of the substrate was measured after 6 week-treatments. Soil samples from three pots per treatment were air-dried and then passed through a 2-mm sieve. For each sample, a soil: water (1:5) suspension was prepared in deionised water and mixed for one hour at 600 u/min, and 21°C. Electric conductivity was measured with a Crison Conductivity-meter 522 and expressed in dS m^-1^.

### Percentage of water content

Part of the fresh material was weighed (fresh weight, FW) before being dried at 65°C, until it reached constant weight and then weighed again (dry weight, DW) and the water content percentage (WC %) was calculated by the following formula:

WC%= [(FW−DW)/FW] x 100  (1)

### Ion content measurements

Potassium and sodium contents were quantified in roots and needles sampled after 6-week treatments. Measurements were performed according to Weimberg [23], in an aqueous extract obtained by heating the sample (0.1 g of dried and ground plant material suspended in 25 ml of water) for 15 min in a boiling water bath, followed by filtration through a filter paper (particle retention 8–12 μm). Sodium and potassium ions were quantified with a PFP7 flame photometer (Jenway Inc., Burlington, USA).

### Osmolyte quantification

Proline (Pro) content was measured in fresh tissue by the ninhydrin-acetic acid method of Bates et al. [24]. Free Pro was extracted in 3% aqueous sulfosalicylic acid; the extract was mixed with one volume of freshly prepared acid ninhydrin and one volume of glacial acetic acid, and incubated at 95°C for 1 h. After cooling on ice, the sample was extracted with two volumes of toluene and the absorbance of the organic phase was determined at 520 nm, using toluene as a blank.

Total soluble sugars (TSS) were quantified according to the technique described by Dubois et al. [25]. Dried material was ground and suspended in 3 mL of 80% methanol, shaken for 20–48 h, and then mixed with sulfuric acid and 5% phenol, before the absorbance of the sample was measured at 490 nm.

### Non-enzymatic antioxidants

The same extract used to quantify total soluble sugars (0.1 g of dried material in 3 mL of 80% methanol), was used to measure total phenolic compounds (TP) and 'total flavonoids' (Fv) (including antioxidant flavonoids but also other phenolics containing a catechol group). TP were quantified according to Blainski et al. [26], by reaction with the Folin-Ciocalteu reagent. Absorbance was measured at 765 nm, and the results expressed in equivalents of gallic acid (mg. eq. GA g^-1^ DW). The antioxidant flavonoids were measured following the method described by Zhishen et al. [27], based on the reaction of catechol groups with AlCl_3_; the absorbance was measured at 510 nm, and the amount of flavonoids was expressed in equivalents of catechin (mg eq C g^-1^ DW).

### Photosynthetic pigments

Total carotenoids (Caro), chlorophyll a (Chl a), and chlorophyll b (Chl b) were quantified following Lichtenthaler and Welburn [28]: 0.1 g fresh material was crushed and shaken in 80% ice-cold acetone for 1 hour on a shaker at 4°C, then centrifuged for 15 min at 3000 x *g*. The supernatant was separated and its absorbance was measured at 663 nm, 646 nm, and 470 nm. The concentration of each group of compounds was calculated according to the following equations, and then converted into μg/g DW:

Chl a (μg/ml)=12.21(A663)− 2.81 (A646) (2)

Chl b (μg/ml) = 20.13 (A646) − 5.03 (A663) (3)

Caro (μg/ml) = (1000 A470 - 3.27 [chl a] - 104 [chl b])/227 (4)

### Statistical analysis

Data were analyzed using SPSS, v. 16 and SYSTAT v.XVI. A Levene test was applied to check whether the requirements of the analysis of variance (ANOVA) were accomplished. The significance of the differences among treatments and among populations was tested by applying a one-way ANOVA, at a confidence level of 95%. When the ANOVA null hypothesis was rejected, posthoc comparisons were performed by the Tukey test. A two-way ANOVA was applied to test the effect of genotype (seedlings' origin), treatments and their interaction on the analysed characteristics. Altitude and main climatic conditions (mean annual temperature, rainfall and evapotranspiration) of the seven populations which were the source of the seeds were correlated to chemical and biochemical characteristics measured in spruce seedlings submitted to stress treatments by the multivariate approach of principal component analysis (PCA). Finally, a linear regression was applied to test the degree of correlation between the analysed characteristics and the electric conductivity in the pots at the end of the treatments.

## Results

### Electric conductivity of the substrate

**Fig 2:**
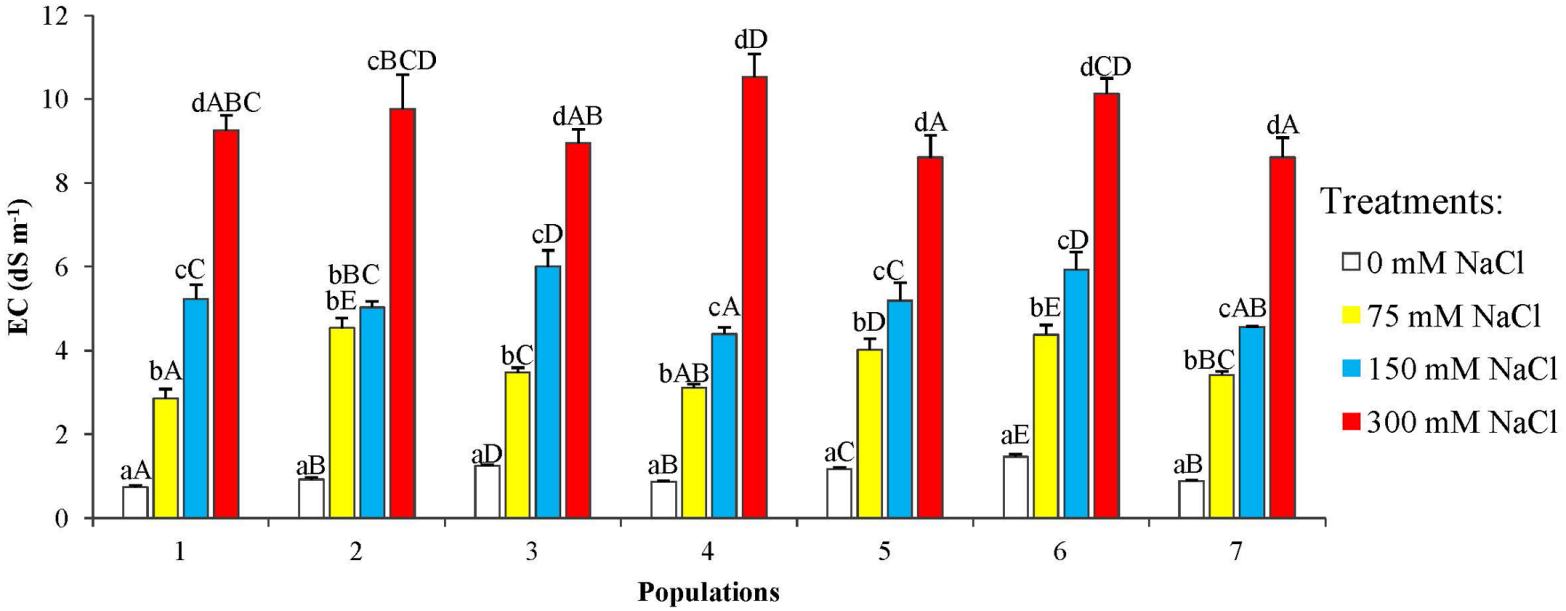
Electric conductivity (EC_1:5_, dS m^-1^) of substrate samples after 6-week treatments with the indicated NaCl concentrations, for the seven *P*. *abies* populations under study. Means with SD (n = 3). For each population, different lowercase letters above the bars indicate significant differences among treatments and different capital letters indicate significant differences among populations undergoing the same treatment, according to the Tukey test (α = 0.05).

Electrical conductivity of the pot substrates increased for all seven studied populations of *P*. *abies* in parallel to the increasing NaCl concentration in the irrigation solutions (Fig 2). The mean substrate EC_1:5_ (for all populations) in the 300 mM NaCl treatment was about 10-fold higher than that registered in the substrate of control plants.

### Percentage of water content

**Fig 3:**
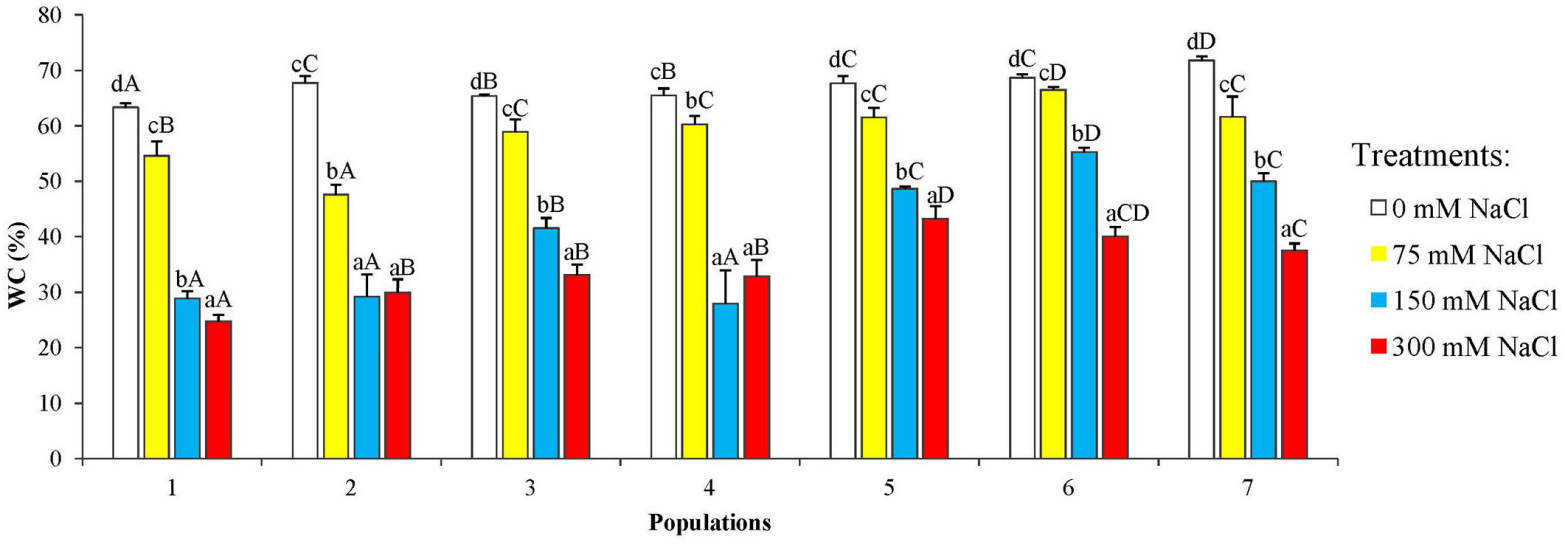
Relative water content (%) in seedlings of the *P. abies* populations under study, after 6-week treatments with the indicated salt concentrations. Means with SD (n = 3). For each population, different lowercase letters above the bars indicate significant differences among treatments and different capital letters indicate significant differences among populations undergoing the same treatment, according to the Tukey test (α = 0.05).

The water content percentage (WC %) in the spruce needles showed a decrease with increasing external salt concentrations, which was statistically significant already in the presence of 75 mM NaCl, the lowest concentration tested (Fig 3). This response was expected, since one of the most general effects of salt stress, due specifically to its osmotic component, is the loss of cellular water. The concentration-dependent reduction in the plants' relative water content was observed in all *P*. *abies* populations; in most cases, WC dropped below 30% in the presence of 300 mM NaCl, yet significant differences among populations were observed for the 75 mM and 150 mM NaCl treatments.

### Photosynthetic pigments

**Fig 4:**
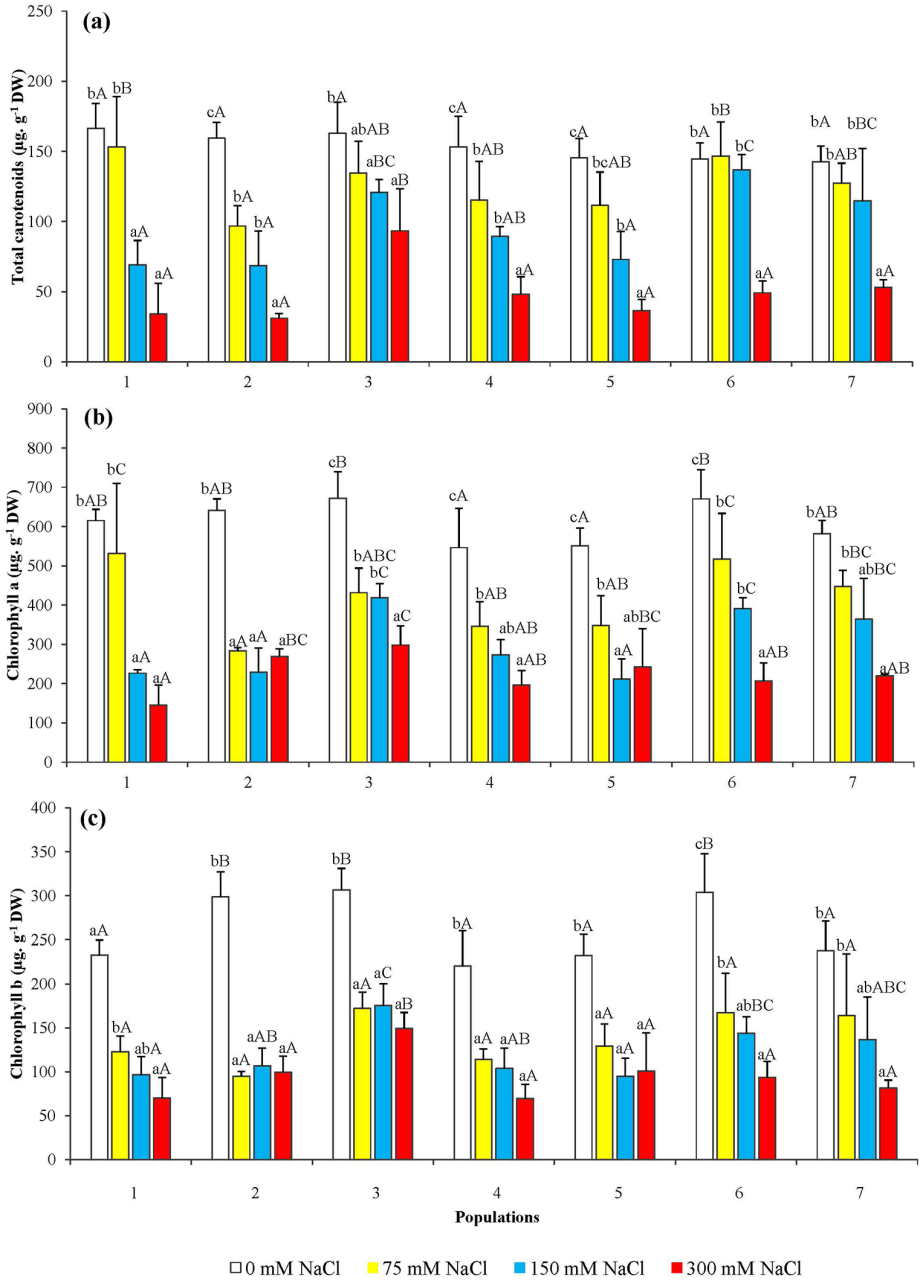
Photosynthetic pigments in the needles of seedlings obtained from the seven *P*. *abies* populations, after 6-week treatments with the indicated NaCl concentrations. Variation in (a) total carotenoids, (b) chlorophyll a and (c) chlorophyll b. Means with SD (n = 3). For each population, different lowercase letters above the bars indicate significant differences among treatments and different capital letters indicate significant differences among populations undergoing the same treatment, according to the Tukey test (α = 0.05).

The photosynthetic pigments measured in the seedling needles showed a reduction with the increase of external salt concentration, for the seven *P*. *abies* populations under study. Total carotenoids (Fig 4a), chlorophyll a (Fig 4b), and chlorophyll b (Fig 4c) decreased with a similar pattern and, in most cases, the differences with the corresponding non-treated controls were found to be significant even in the presence of 75 mM NaCl, the lowest salt concentration applied in our experiments. Here again, quantitative differences in the response to salt stress, regarding the concentration-dependent reduction in photosynthetic pigments, were observed among populations.

**Fig 5:**
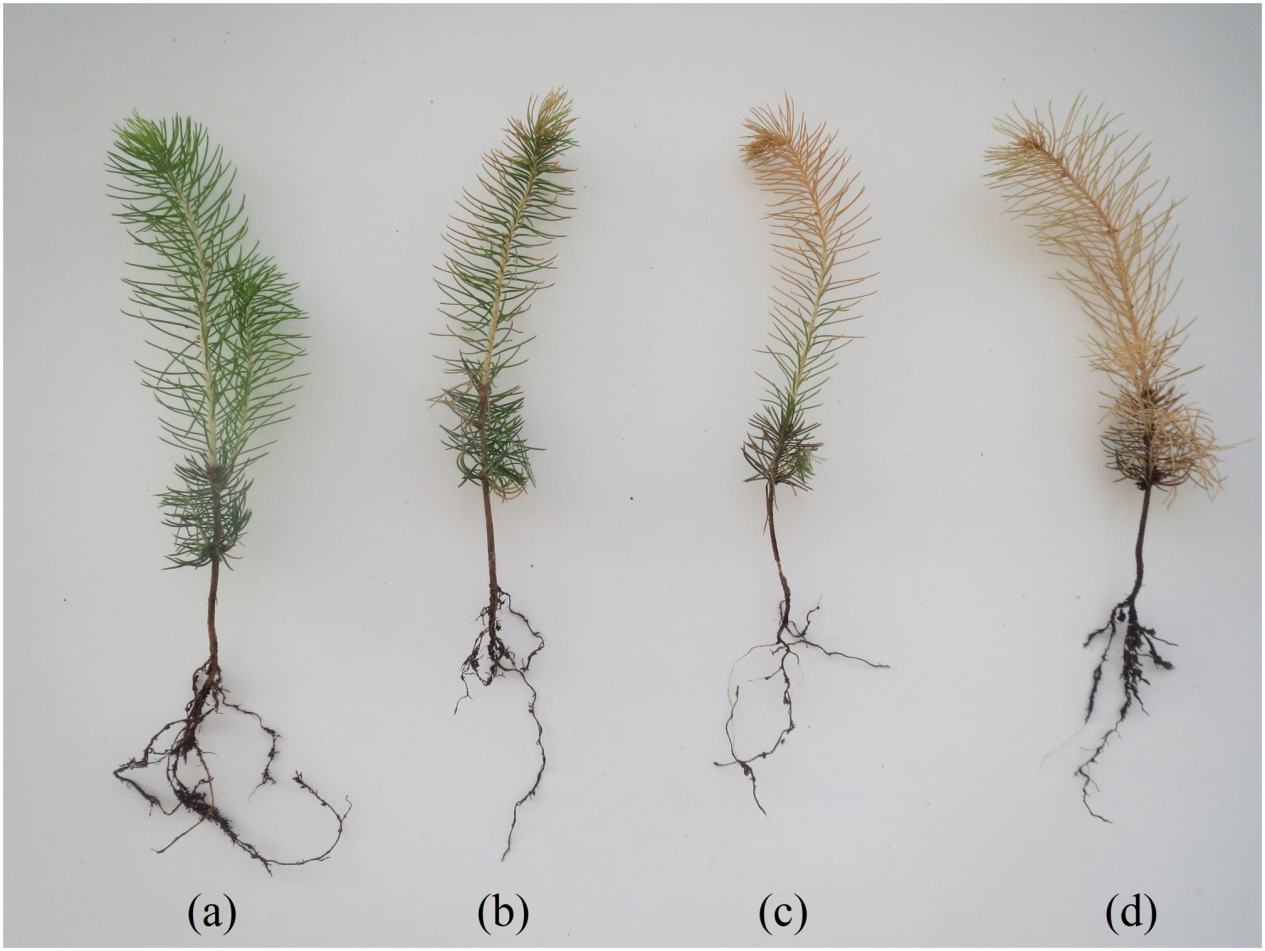
Visual effect of salt treatments on *P*. *abies* seedlings. Treatments: (a) control, (b) 75 mM, (c) 150 mM and (d) 300 mM NaCl.

The reduction in pigments levels was reflected in a visible change in the needle colour, from green to yellow-reddish, in all studied populations. This change started at the needle tips, and was more evident as the NaCl concentration increased; an example is shown in Fig 5.

### Monovalent cation levels

**Fig 6:**
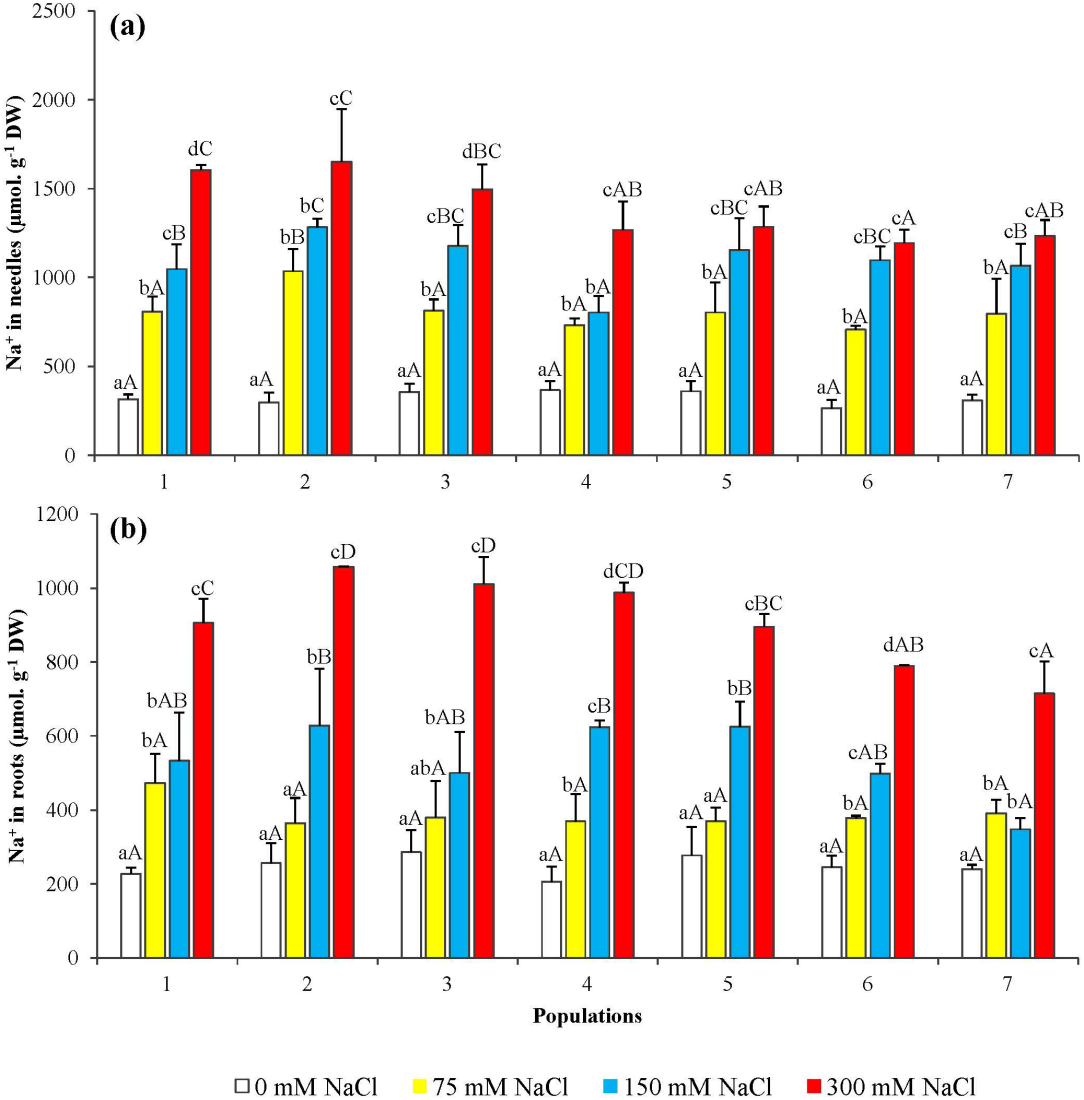
Sodium ion (Na^+^) levels in seedlings from the seven *P. abies* populations, after 6-week treatments with the indicated NaCl concentrations. Changes in (a) needle and (b) root Na^+^ contents. Means with SD (n = 3). For each population, different lowercase letters above the bars indicate significant differences among treatments and different capital letters indicate significant differences among populations undergoing the same treatment, according to the Tukey test (α = 0.05).

**Fig 7:**
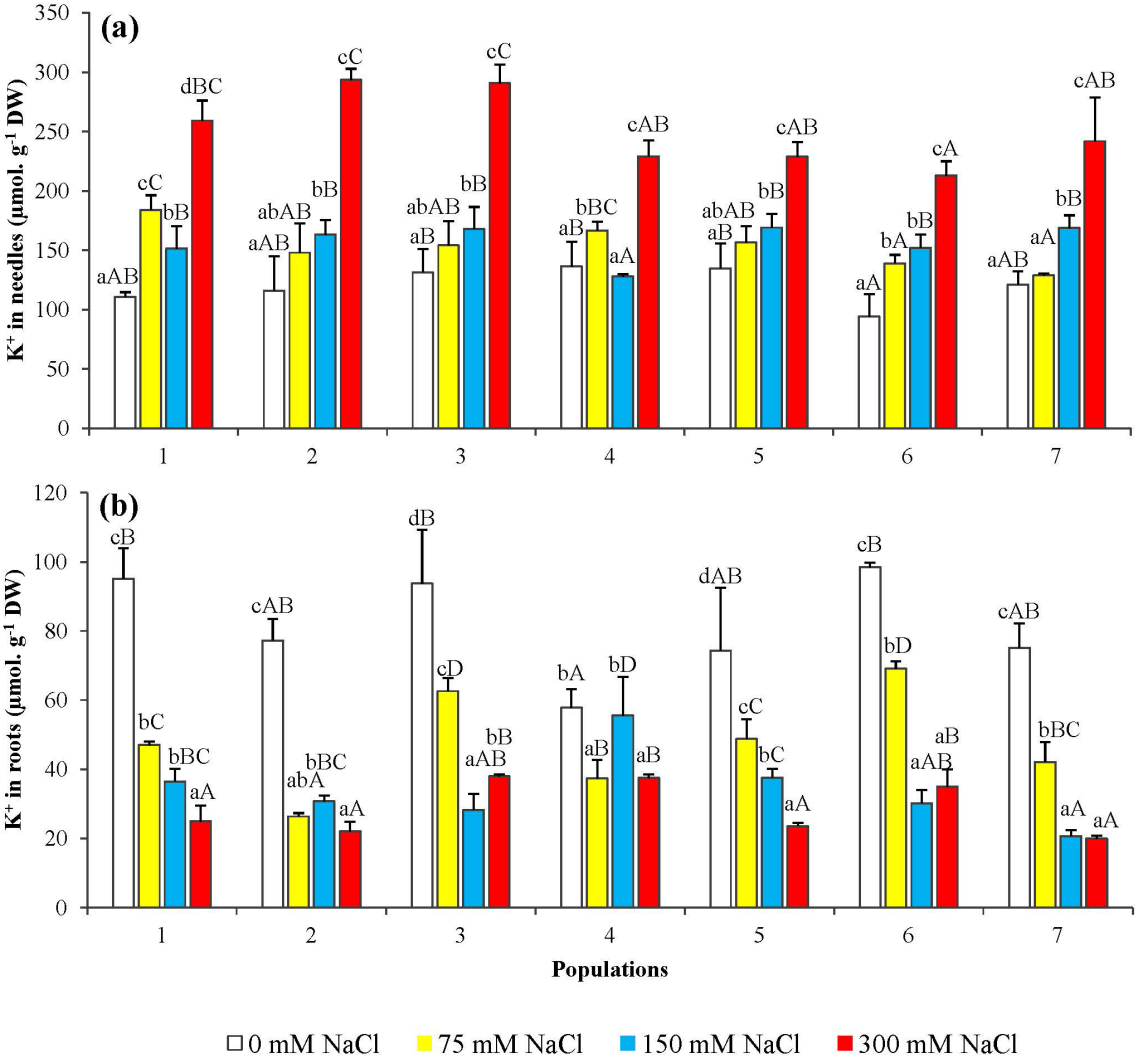
Potassium ion (K^+^) levels in seedlings from the seven *P*. *abies* populations, after 6-week treatments with the indicated NaCl concentrations. Changes in (a) needle and (b) root K^+^ contents. Means with SD (n = 3). For each population, different lowercase letters above the bars indicate significant differences among treatments and different capital letters indicate significant differences among populations undergoing the same treatment, according to the Tukey test (α = 0.05).

Sodium contents increased in both, the needles and the roots of all salt-treated seedlings in parallel to the increase of the concentration of NaCl in the irrigation solution (Fig 6). However, the absolute Na^+^ levels reached in salt-treated seedlings were somewhat higher in the needles (about 1.5 mmol g^-1^ DW, as average, in the presence of 300 mM NaCl) (Fig 6a) than in the roots (below 1.0 mmol g^-1^ DW for most populations, at the same external salt concentration) (Fig 6b). Potassium levels also increased in the needles upon salt treatment of the *P*. *abies* seedlings (Fig 7a), reaching up to ca. 3-fold over the controls for some populations, in the presence of 300 mM NaCl. Contrary to what was observed in the needles, K^+^ contents showed a significant salt concentration-dependent decrease in the roots (Fig 7b).

**Fig 8:**
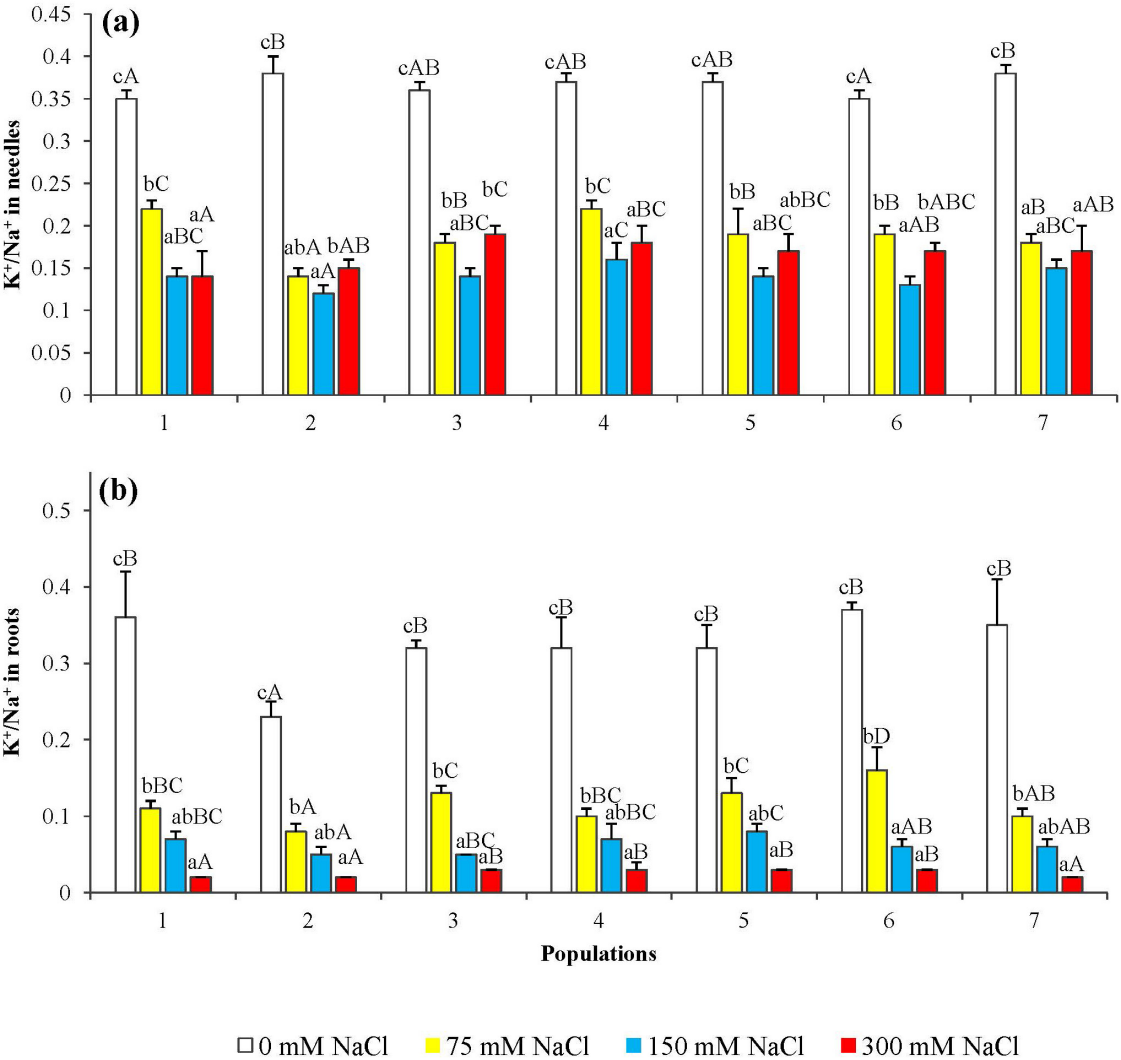
Potassium over sodium ratio (K^+^/Na^+^) in seedlings from the seven *P. abies* populations, after 6-week treatments with the indicated NaCl concentrations. in (a) needles and (b) roots. Means with SD (n = 3). For each population, different lowercase letters above the bars indicate significant differences among treatments and capital letters indicate significant differences among populations undergoing the same treatment, according to the Tukey test (α = 0.05).

The ratio K^+^/Na^+^, calculated from the aforementioned data, generally decreased with increasing NaCl concentrations, both in roots and needles and in plants from all populations, except that a small—but statistically significant—increase in K^+^/Na^+^ ratios was observed in the needles of plants from populations 2, 3, and 6 at the highest salt concentration tested (300 mM NaCl), as compared to the 150 mM NaCl treatment (Fig 8).

### Osmolyte contents

**Fig 9:**
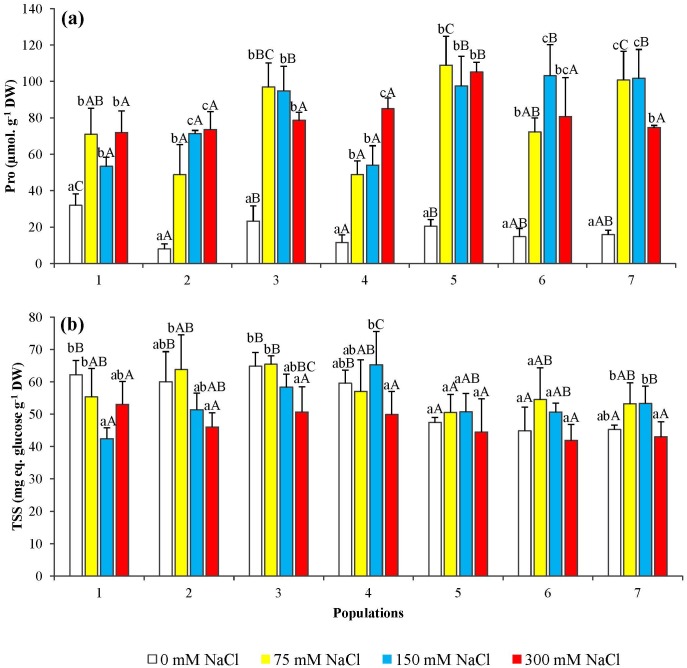
Variation of osmolyte content in seedlings from the seven *P*. *abies* populations, after 6-week treatments with the indicated NaCl concentrations. Changes in (a) proline (Pro) and (b) total solube sugars (TSS). Means with SD (n = 3). For each population, different lowercase letters above the bars indicate significant differences among treatments and capital letters indicate significant differences among populations undergoing the same treatment, according to the Tukey test (α = 0.05).

Proline contents in the needles of all seedlings tested showed a significant salt-induced increase, even at the lowest NaCl concentration used (75 mM), although quantitative differences were found between different populations; in some of them, maximum Pro levels up to about 100 μmol g^-1^ DW were measured in the presence of 300 mM NaCl (Fig 9a).

No clear pattern of variation was detected in total soluble sugar contents in the needles of salt-treated seedlings, except for a slight decreasing trend observed at the highest NaCl concentrations; nevertheless, in most cases the differences were not statistically significant (Fig 9b).

### Non-enzymatic anti-oxidants

**Fig 10:**
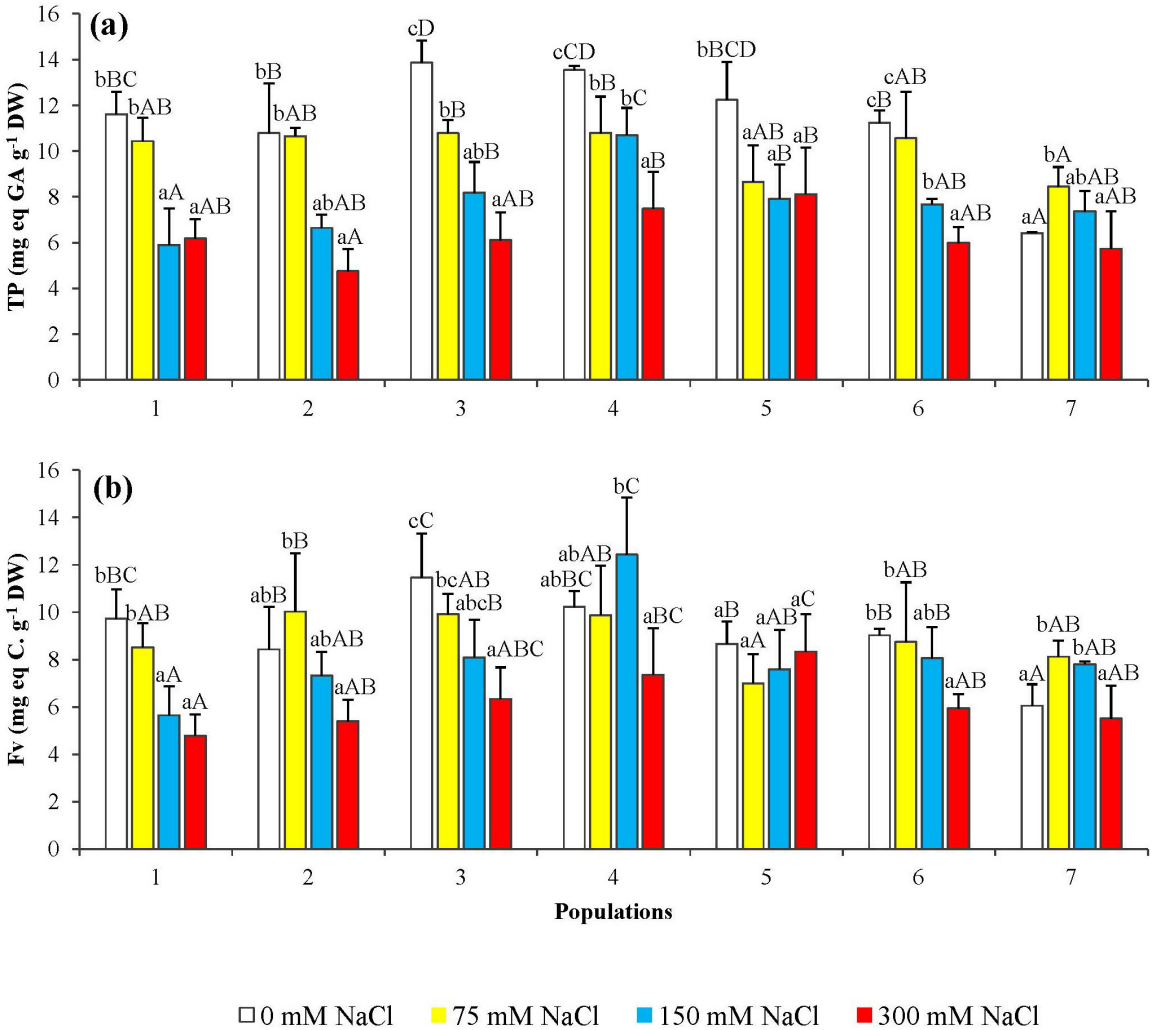
Total phenolic compounds (TP) (a) and total flavonoids (Fv) (b) levels in seedlings from the seven *P*. *abies* populations, after 6-week treatments with the indicated NaCl concentrations. Means with SD (n = 3). For each population, different lowercase letters above the bars indicate significant differences among treatments and capital letters indicate significant differences among populations undergoing the same treatment, according to the Tukey test (α = 0.05).

Total phenolic compounds (Fig 10a) and total flavonoids (Fig 10b) were quantified in the needles of salt-treated seedlings. A significant, concentration-dependent decrease in the contents of these secondary metabolites was observed, with a nearly uniform pattern in all studied *P*. *abies* populations.

### Statistical analysis of the results

The one-way ANOVA performed for treatments within one population and for populations within one treatment indicated significant differences. In addition, a two-way ANOVA was carried out to assess the influence of both, seedling origin and treatment on the biochemical parameters under study. As shown in Table 2, for all studied markers there are significant differences, at the 95% confidence level, according to 'treatment' and to 'population', except for Na^+^ in roots concerning the 'population' factor. The interactions of the two factors are also significant, with the exception of Chl b, and root contents of Na^+^ and K^+^.

**Table 2:** Effect of population and treatment on the measured biomarkers as established by two-way ANOVA. Values represent P and its significance at 95% confidence level.

Biomarker	Population	Treatment	Interaction
**Pro**	**0.0000**	**0.0000**	**0.0000**
**TSS**	**0.0001**	**0.0000**	**0.0474**
**TP**	**0.00000**	**0.0000**	**0.0053**
**Fv**	**0.0001**	**0.0000**	**0.0015**
**Caro**	**0.0000**	**0.0000**	**0.0055**
**Chl a**	**0.0000**	**0.0000**	**0.0020**
**Chl b**	**0.0001**	**0.0000**	**0.1425**
**WC**	**0.0000**	**0.0000**	**0.0000**
**Na**_**r**_	**0.0666**	**0.0000**	**0.6666**
**Na**_**n**_	**0.0000**	**0.0000**	**0.0033**
**K**_**r**_	**0.0206**	**0.0000**	**0.4847**
**K**_**n**_	**0.0213**	**0.0000**	**0.0504**

Abbreviations: proline (Pro), total sugars (TSS), total phenolics (TP), antioxidant flavonoids (Fv), carotenoids (Caro), chlorophyll a and b (Chl a and Chl b), water content (WC%), sodium in roots and needles (Na_r_ and Na_n_), potassium in roots and needles (K_r_ and K_n_).

**Fig 11:**
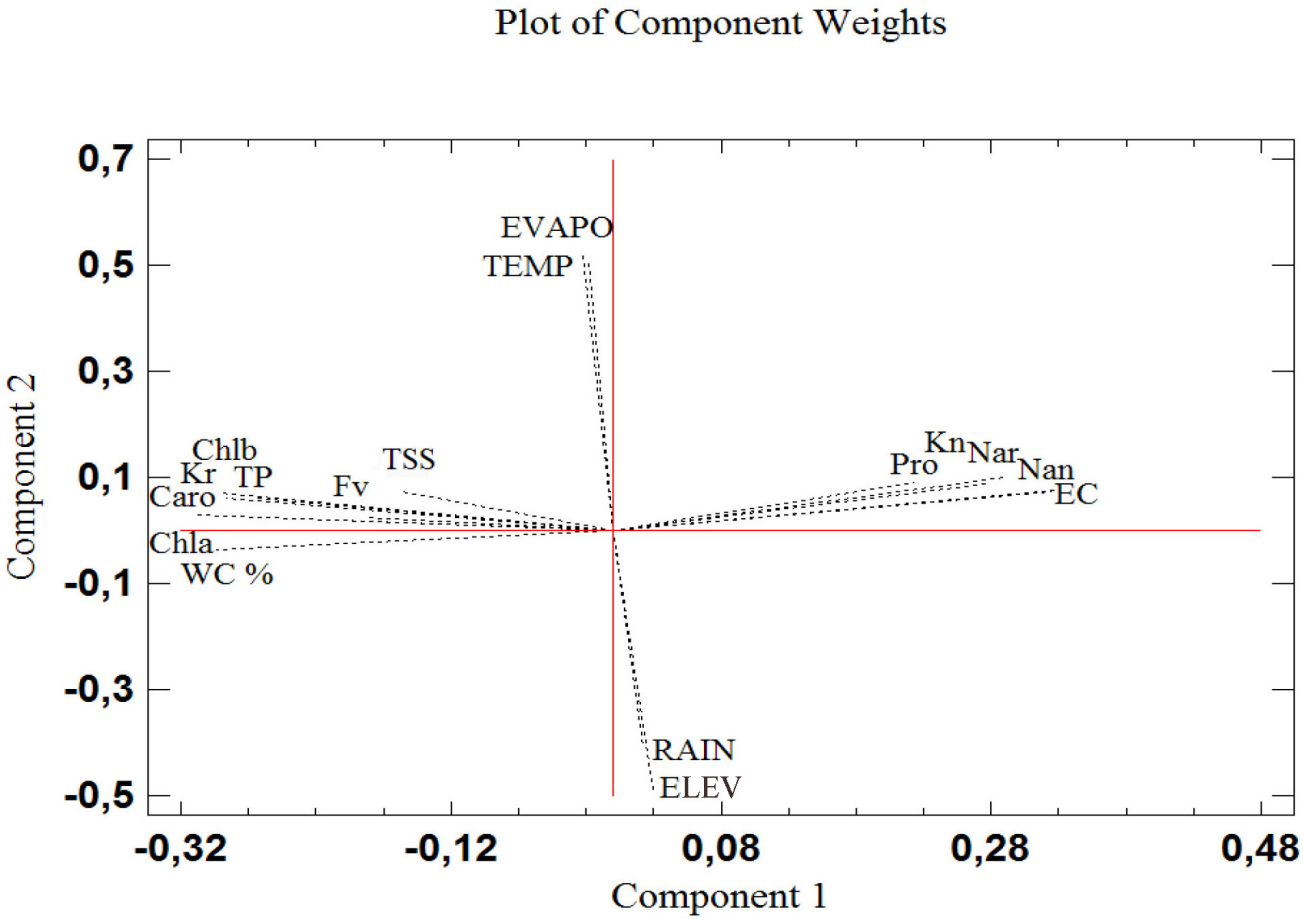
PCA of stress markers measured in *P*. *abies* seedlings, in correlation to EC of the substrate at the end of treatments and parental populations' altitude and climatic variables. Abbreviations: proline (Pro), total sugars (TSS), total phenolics (TP), antioxidant flavonoids (Fv), carotenoids (Caro), chlorophyll a and b (Chla and Chlb), water content (WC%), sodium in roots and needles (Na_r_ and Na_n_), potassium in roots and needles (K_r_ and K_n_), altitude (ELEV), mean annual temperature (TEMP), rainfall (RAIN) and evapotranspiration (EVAPO).

The biochemical parameters measured in seedlings were subjected to a principal component analysis (PCA), together with the soil electrical conductivity in the pots measured at the end of the treatments, which reflects the intensity of the salt stress applied to the plants; environmental variables at the origin sites of seeds (altitude, mean annual temperature, mean annual rainfall and mean annual evapotranspiration) were also included in the analysis. Three components with an Eigenvalue equal to or greater than 1 explained a cumulative percentage of variance of 80%. The plot of the loading vectors shows the relationship between variables (Fig 11). The first component (X-axis), which explained 49% of variance, is clearly determined by the electric conductivity of the substrates. The loading vectors of the biochemical variables presented very small angles with the X-axis, indicating a highly significant correlation with salinity, either positive—for those variables increasing in parallel with increasing external NaCl concentrations (proline, Na^+^ in needles and roots and K^+^ in needles)–or negative (for relative water content, and the level of carotenoids, chlorophyll a and b, total sugars, total phenolics and flavonoids and K^+^ in roots). The second component (Y axis), which explained a further 21% of variance, is related to the environmental conditions. As expected, evapotranspiration and temperature were positively correlated with each other, with the corresponding vectors close to the positive part of the Y-axis, but negatively with altitude and rainfall. However, no correlation was found between the biochemical variables in plants and the average climatic conditions at the origin sites of the seeds.

Additionally, a regression analysis was performed to check the relation between the salt stress treatments (soil EC) and the biochemical stress markers measured in the plants (Table 3). Changes in all selected biomarkers, either increasing or decreasing their contents in the spruce seedlings, showed significant correlations with the degree of applied salt stress when all plants were considered together. The highest coefficients of determination (R^2^ > 0.9) were calculated for total phenolic compounds, carotenoids, Na^+^ contents in needles and roots, and K^+^ contents in needles. Somewhat lower, but still highly significant R squared values (R^2^ > 0.85) were observed for flavonoid and chlorophyll a contents. When this analysis was applied independently to each population, quantitative differences were observed, probably reflecting genetic variability among populations; nevertheless, in most cases the same relations were maintained, indicating that parameters such as total phenolic compounds and carotenoid contents, and accumulation in needles of Na^+^ or K^+^ ions may be employed as reliable biomarkers of salt stress in *Picea abies* seedlings.

**Table 3:** Coefficients of determination (R^2^) established by linear regression between seedlings' characteristics and electric conductivity at the end of the treatments. Biomarker abbreviations as in Table 2.

R^2^								
Biomarker	Populations							All seedlings seedlings
	1	2	3	4	5	6	7	
**Pro**	**0.54**	**0.75**	**0.29**	**0.95**	**0.51**	**0.47**	**0.23**	**0.63**
**TSS**	**0.24**	**0.71**	**0.87**	**0.33**	**0.25**	**0.13**	**0.08**	**0.73**
**TP**	**0.71**	**0.85**	**0.93**	**0.96**	**0.59**	**0.94**	**0.16**	**0.93**
**Fv**	**0.86**	**0.52**	**0.96**	**0.09**	**0.13**	**0.92**	**0.09**	**0.86**
**Caro**	**0.86**	**0.93**	**0.98**	**0.98**	**0.96**	**0.79**	**0.94**	**0.99**
**Chla**	**0.84**	**0.52**	**0.86**	**0.87**	**0.66**	**0.98**	**0.98**	**0.88**
**Chlb**	**0.79**	**0.52**	**0.67**	**0.81**	**0.64**	**0.84**	**0.95**	**0.75**
**Na**_**r**_	**0.99**	**0.95**	**0.93**	**0.98**	**0.96**	**0.99**	**0.94**	**0.98**
**Na**_**n**_	**0.99**	**0.91**	**0.94**	**0.98**	**0.86**	**0.86**	**0.87**	**0.93**
**K**_**r**_	**0.78**	**0.61**	**0.64**	**0.35**	**0.92**	**0.71**	**0.75**	**0.78**
**K**_**n**_	**0.84**	**0.92**	**0.91**	**0.63**	**0.97**	**0.99**	**0.93**	**0.93**
**WC%**	**0.81**	**0.74**	**0.91**	**0.63**	**0.89**	**0.93**	**0.97**	**0.87**

## Discussion

The first and most general reaction of plants to different stress conditions is the inhibition of growth, as they redirect all their resources (energy, metabolic precursors) from normal primary metabolism and biomass accumulation to the defence reactions against stress [29]. Therefore, morphological stress markers based on growth parameters are extremely reliable and commonly used to assess the effects of stress in most plant species. However, they are not so useful for species of slow growth, such as forest trees; in these cases it is especially important to define appropriate biochemical or molecular stress markers which may help to quickly detect and minimise the deleterious effects of abiotic stress on the plants.

All plants activate a series of basic, conserved mechanisms as a response to environmental stressful conditions such as, for example, drought, cold, high temperatures or high soil salinity; they include, among others, the control of ion transport, protection of the photosynthetic machinery, maintenance of cellular osmotic balance or activation of antioxidant systems [14,30–32]. Several metabolites involved in these responses—mono and divalent cations, osmolytes (proline, glycine betaine, and soluble sugars), photosynthetic pigments, antioxidant compounds, etc.–can be used, *a priori*, as biochemical stress markers. Norway spruce represents a good example of a salt-sensitive, slow-growth forest species for which it would be useful to define reliable salt stress biomarkers. They would contribute to the correct management of spruce populations exposed to the risk of increased salinity, allowing the rapid analysis of the level of salt stress the trees may suffer in their natural stands.

Under the conditions of the present study, with relatively short salt treatments (six weeks), we could not detect significant changes in growth parameters, apart from a reduction in the water content of needles, even in the seedlings maintained in the presence of 300 mM NaCl. Nevertheless, longer salt treatments (four months) at lower salt concentrations (up to 120 mM NaCl), indeed inhibit growth of spruce seedlings, as we observed in previous experiments, independent of those presented here—although using the same batches of seeds (S1 Fig).

In the present work, we report a wide screening for biochemical markers of salinity stress in Norway spruce. There is at present very little information on this topic, which has not attracted much attention since the natural habitats of this species are not saline. Yet, many trees may indeed be affected by salt in the vicinity of mountain roads due to the common practice of de-icing in winter using large amounts of NaCl. We have worked with seedlings obtained by germination of seeds from seven different Romanian populations of *Picea abies*, to define those compounds that could be used as general salt stress biomarkers, independently of the particular genetic background of the parental populations of the seeds. Changes in the levels in plants of all investigated biochemical stress markers showed a generally good correlation, either positive or negative, with variations of substrate EC; that is, with the intensity of salt stress. No correlation was found with the environmental variables of the spruce populations from which the seeds were obtained; this was to be expected, since natural stressful conditions in the spruce habitats do not include high soil salinity, and there was no possibility of 'adaptation' to salty conditions. However, these correlations were statistically more significant for some biomarkers, such as total phenolic compounds, total carotenoids or Na^+^ and K^+^ levels in needles, which in the linear regression analyses showed higher coefficients of determination (R^2^) than, for example, proline or total soluble sugars. It should also be mentioned that, when these analyses were performed independently for each population, quantitative differences were found among them, although maintaining the general qualitative trends.

Potassium, as the 'physiological' cation, is essential for plant growth and development, and K^+^ deficiency has negative effects on photosynthesis, osmoregulation, protein biosynthesis and turgor driven movements [33]. High Na^+^ concentrations, on the contrary, inhibit many enzymatic activities and physiological processes, as well as interfere with K^+^ uptake and homeostasis, as both cations compete for the same transport systems [34,35]. Consequently, Na^+^ accumulation in the plants in the presence of high external salt concentrations is generally accompanied by a reduction in K^+^ levels and K^+^/Na^+^ ratios. In our experiments we have observed, indeed, Na^+^ accumulation in *P*. *abies* needles and roots, but K^+^ contents actually increased in the needles (although decreased in roots, as expected). This behaviour can be interpreted as a defence mechanism that, by activating K^+^ transport from the roots to the plant aerial parts, avoids excessively low K^+^/Na^+^ ratios in the needles. Sodium accumulation in the roots and needles of *Picea abies* and other spruce species, as a response to salt treatments, has already been reported [36,37]. Salt can be taken up by both roots and needles, as shown by irrigation with NaCl and spraying on spruce foliage [38]. On the other hand, little information is available regarding changes of potassium contents in coniferous species, in response to salinity. For example, low salt treatments (30 mM NaCl) on 6 month-old tamarack (*Larix laricina*) caused a decrease of K^+^ in roots, a reduction in chlorophyll a, b and carotenoid levels in needles, and accumulation of Na^+^ predominantly in roots [39]. Maintaining relatively high cytosolic K^+^/Na^+^ ratios is of paramount importance for the ability of plants to survive the salt stress [40]. *Thellungiella halophila*, a salt-tolerant relative of the glycophyte *Arabidospis*, is able to increase its mesophyll K^+^ content under saline conditions, whereas the model species shows a clear decline [41]. Despite their assumed important role in the mechanisms of salt tolerance, only a small number of genes responsible for Na^+^ and K^+^ transport have been fully characterized. The majority of such studies have been carried out on *Arabidospi*s, and at present practically nothing is known about these processes in coniferous species. Yet it is to be expected that the characterization of genes involved in ion transport—and other stress responses—will be facilitated in the near future, since the first whole-genome draft sequence of the Norway spruce is already available, although not yet annotated [42] and micro-array transcript profiles have been recently assembled in a data base called PiceaGenExpress [43]. This will help to establish how the complex regulatory mechanisms of Na^+^ and K^+^ uptake and distribution in plants, mediated by several transport systems [35,44], operate in *Picea abies*.

Carotenoids, which may also be used as reliable stress biomarkers in this species, are multifunctional compounds that serve as accessory light harvesting pigments, transferring energy from solar emissions to chlorophylls and thus extending the range of light wavelengths for photosynthesis; they also function protecting chlorophylls from destructive photooxidation reactions and scavenging singlet oxygen [45,46–48]. Due to their antioxidant properties, carotenoids could probably contribute to the responses of plants to salinity, as indicated by the enhanced salt resistance of transgenic plants engineered to accumulate increased concentrations of these compounds, by overexpression of the appropriated genes of the carotenoid biosynthesis pathway [49–51]. Here again, there are not yet data on the expression of these genes in spruce, but it is known that in other glycophytes, for example in tomato, salt stress inhibits the expression of major genes involved in carotenoid biosynthesis [52]. Consequently, there are numerous reports of decreased levels of total carotenoids at high salinities in different plant taxa, including trees [53–56].

In the present work, we have detected significant, concentration-dependent reductions in the contents of photosynthetic pigments (chlorophylls a and b, and total carotenoids) in NaCl-treated *P*. *abies* seedlings. This effect could be visually observed by a change in the needles colour. Similar damage caused by salt stress has been previously reported for needles of other *Pinaceae* family species [57,58].

Phenolic compounds—and especially a subgroup of them, the flavonoids—are plant secondary metabolites which are important in the mechanisms of adaptation to abiotic stresses [59], among many other biological functions. Since many flavonoids and other phenolic compounds are strong antioxidants, their accumulation in plants can reduce the oxidative damage induced by different abiotic stresses, including high salinity [60]. Accordingly, concentration-dependent, salt-induced increases in antioxidant phenolics levels have been reported for different plant species [61,62]. In *P*. *abies* seedlings, however, a significant reduction of total phenolic compounds and antioxidant flavonoids was observed as a response to the salt treatments. This reduction is probably related to phenolics functions other than their possible participation as antioxidants in stress defence mechanisms. In any case, concerning the aim of this study, it is important to point out the high (negative) correlation of total phenolics contents with the NaCl concentration applied to the spruce seedlings, supporting the use of these compounds as salt stress biomarkers in *P*. *abies*.

The contents in the spruce seedlings of these biochemical markers cannot be directly compared with the growth parameters previously determined in salt-stressed plants (data included as supporting information), since the experiments were performed independently with different individuals—although grown from the same seed batches—and under different conditions. However, when establishing statistical correlations between salt treatments and the biomarkers, the calculated coefficients of determination (R^2^) were similarly high (S1 Table). Yet, determining changes in the levels of biochemical compounds in response to stress, instead of measuring plant growth parameters, is advantageous in species with slow growth rates, such as conifers. Such simple biochemical markers are equally useful to estimate the degree of stress affecting the trees in their natural stands. On the other hand, we could not identify any population more salt tolerant than the others, or showing higher statistical correlations of all salt stress biomarkers with the degree of stress. Therefore, the choice of parental material does not influence significantly the response of spruce seedlings to salt stress, at least in the case of the seven populations analysed here.

In conclusion, we propose that—from a practical point of view—the (decreasing) levels of total phenolics or total carotenoids, and the (increasing) levels of Na^+^ or K^+^ ions, in *P*. *abies* needles, should be considered as the most reliable biomarkers for salt stress in this species, at least at the seedling stage. They all show very high correlation with the intensity of salt stress, for most if not all populations, so that they could be used independently of the genetic background of the seeds parental population. In addition, there are relatively easy, quantitative and non-destructive assays to determine all these compounds, requiring simple equipment and little amount of plant material.

## Supporting Information

S1 Fig Effect of four months salt treatments on growth in *P*. *abies* seedlings. Treatments performed on peat substrate under controlled conditions (mean temperature 21°C, relative humidity 50%). Control seedlings were watered two times a week with tap water using a volume of 200 mL/pot (L20xW10xH20 cm), while for salt treatments the same volume was used but containing the corresponding NaCl concentrations. Means with SD (n = 5). For each population, different lowercase letters above the bars indicate significant differences among treatments and capital letters indicate significant differences among populations undergoing the same treatment, according to the Tukey test (α = 0.05).
(TIF)

S1 Table Coefficients of determination (R^2^) established by linear regression between seedlings' growth traits and NaCl treatments. Abbreviations. SL: stem length; RL: root length; SFM: seedling fresh mass; NN: number of needles.
(DOCX)
