# Supplementary figures and images for: Identification of Salt Stress Biomarkers in Romanian Carpathian Populations of Picea abies (L.) Karst
Source: PLoS One. 2015 Aug 19;10(8):e0135419. doi: 10.1371/journal.pone.0135419 (PMC4545727; doi:10.1371/journal.pone.0135419)

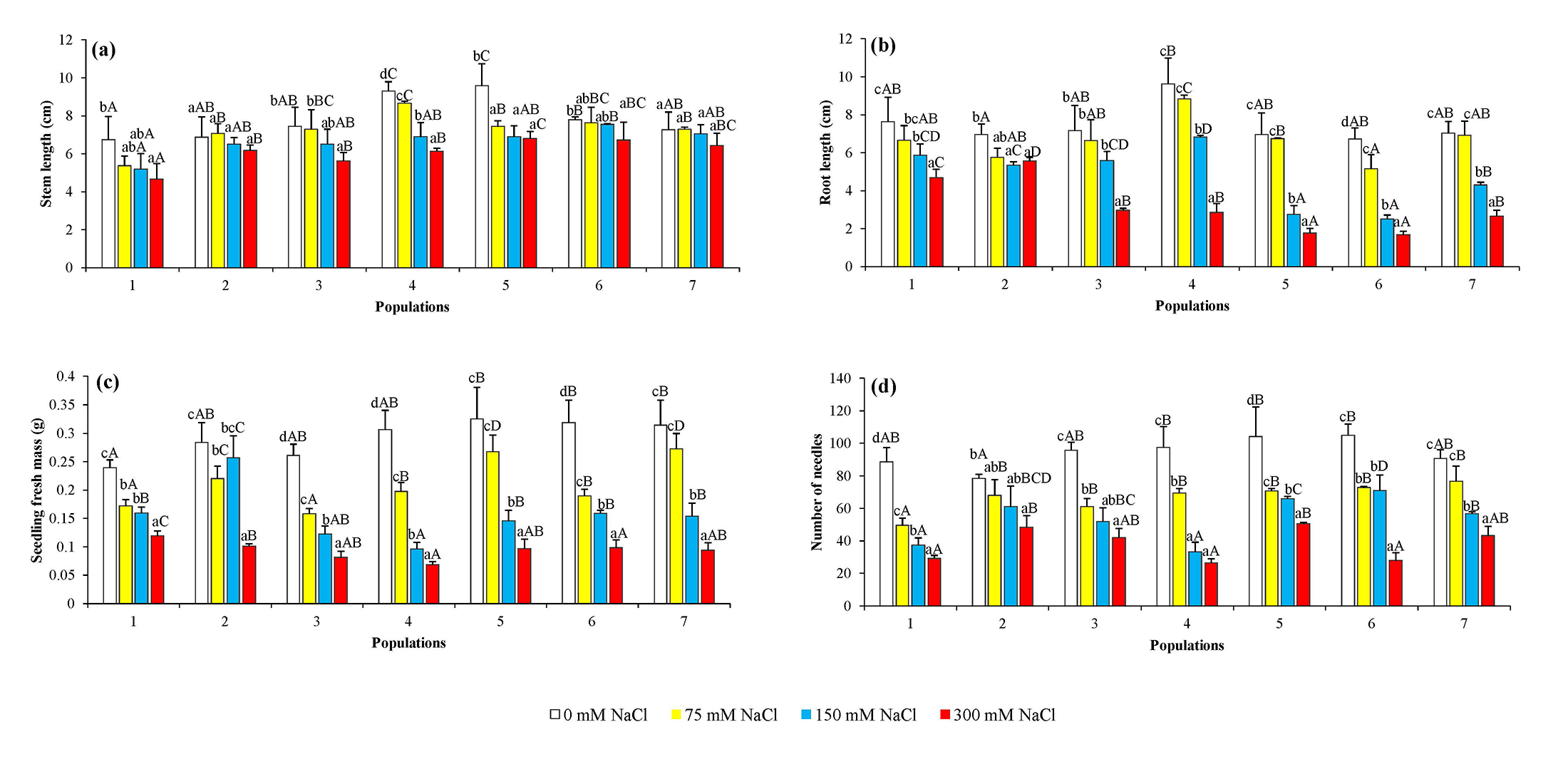

Supplement: S1 Fig — Treatments performed on peat substrate under controlled conditions (mean temperature 21°C, relative humidity 50%). Control seedlings were watered two times a week with tap water using a volume of 200 mL/pot (L20xW10xH20 cm), while for salt treatments the same volume was used but containing the corresponding NaCl concentrations. Means with SD (n = 5). For each population, different lowercase letters above the bars indicate significant differences among treatments and capital letters indicate significant differences among populations undergoing the same treatment, according to the Tukey test (α = 0.05). (TIF) [file pone.0135419.s001.tif]
