# Supplementary material for: Identification of Salt Stress Biomarkers in Romanian Carpathian Populations of Picea abies (L.) Karst
Source: PLoS One. 2015 Aug 19;10(8):e0135419. doi: 10.1371/journal.pone.0135419 (PMC4545727; doi:10.1371/journal.pone.0135419)
Supplement: S1 Table — Abbreviations. SL: stem length; RL: root length; SFM: seedling fresh mass; NN: number of needles. (DOCX) [file pone.0135419.s002.docx]

**S1 Table.** **Coefficients of determination (R^2^) established by linear regression between seedlings' growth traits and NaCl treatments.**

| **R^2^** | | | | | | | | |
| --- | --- | --- | --- | --- | --- | --- | --- | --- |
| **Biomarkers** | **Populations** | | | | | | | **All Seedlings** |
|  | 1 | 2 | 3 | 4 | 5 | 6 | 7 |  |
| **SL** | 0.78 | 0.8 | 0.97 | 0.91 | 0.63 | 0.92 | 0.89 | 0.94 |
| **RL** | 0.98 | 0.51 | 0.97 | 0.97 | 0.83 | 0.88 | 0.91 | 0.93 |
| **SFM** | 0.9 | 0.8 | 0.84 | 0.83 | 0.9 | 0.84 | 0.92 | 0.95 |
| **NN** | 0.75 | 0.98 | 0.78 | 0.83 | 0.82 | 0.94 | 0.94 | 0.91 |

Abbreviations. SL: stem length; RL: root length; SFM: seedling fresh mass; NN: number of needles.
